# Supplementary material for: Resolving the full spectrum of human genome variation using Linked-Reads
Source: Genome Res. 2019 Apr;29(4):635–45. doi: 10.1101/gr.234443.118 (PMC6442396; doi:10.1101/gr.234443.118)
Supplement: Supplemental Material [file supp_gr.234443.118_Supplemental_longranger-2.2.zip › longranger-2.2.tar.bz2!/lr2.2-public/lib/stan/cmdstan/stan/doc/api/html/namespacemembers_b.html]

Stan: Namespace Members


|  |  |
| --- | --- |
|  | Stan  2.5.0  probability, sampling & optimization |


- Main Page
- Related Pages
- Namespaces
- Classes
- Files

- Namespace List
- Namespace Members

- All
- Functions
- Variables
- Typedefs
- Enumerations
- Enumerator

- a
- b
- c
- d
- e
- f
- g
- h
- i
- j
- l
- m
- n
- o
- p
- q
- r
- s
- t
- u
- v
- w


All Classes Namespaces Files Functions Variables Typedefs Enumerations Enumerator Friends Macros Pages

Here is a list of all namespace members with links to the namespace documentation for each member:

### - b -

- base\_expr\_type
  : stan::gm
- bernoulli\_ccdf\_log()
  : stan::prob
- bernoulli\_cdf()
  : stan::prob
- bernoulli\_cdf\_log()
  : stan::prob
- bernoulli\_log()
  : stan::prob
- bernoulli\_logit\_log()
  : stan::prob
- bernoulli\_rng()
  : stan::prob
- bessel\_first\_kind()
  : stan::agrad
  , stan::math
- bessel\_second\_kind()
  : stan::agrad
  , stan::math
- beta\_binomial\_ccdf\_log()
  : stan::prob
- beta\_binomial\_cdf()
  : stan::prob
- beta\_binomial\_cdf\_log()
  : stan::prob
- beta\_binomial\_log()
  : stan::prob
- beta\_binomial\_rng()
  : stan::prob
- beta\_ccdf\_log()
  : stan::prob
- beta\_cdf()
  : stan::prob
- beta\_cdf\_log()
  : stan::prob
- beta\_log()
  : stan::prob
- beta\_rng()
  : stan::prob
- binary\_log\_loss()
  : stan::agrad
  , stan::math
- binary\_op\_f
  : stan::gm
- binomial\_ccdf\_log()
  : stan::prob
- binomial\_cdf()
  : stan::prob
- binomial\_cdf\_log()
  : stan::prob
- binomial\_coefficient\_log()
  : stan::agrad
  , stan::math
- binomial\_log()
  : stan::prob
- binomial\_logit\_log()
  : stan::prob
- binomial\_rng()
  : stan::prob
- block()
  : stan::math
- bool\_argument
  : stan::gm


---

[ Stan Home Page ]

*© 2011–2014,
Stan Development Team.*
